# Red Cell Distribution Width-to-Platelet Ratio and Other Hematological Markers as Early Predictors of Bronchopulmonary Dysplasia in Preterm Infants

**DOI:** 10.3390/children12091215

**Published:** 2025-09-10

**Authors:** Baran Cengiz Arcagok, Ibrahim Kandemir

**Affiliations:** 1 Department of Pediatrics, Division of Neonatology, School of Medicine, Acibadem University, Istanbul 34752, Turkey; 2 Department of Pediatrics, Istanbul Health and Technology University, Istanbul 34275, Turkey; ibrahim.kandemir@istun.edu.tr

**Keywords:** bronchopulmonary dysplasia, preterm infants platelet-to-lymphocyte ratio, RDW-to-platelet ratio, inflammatory biomarkers

## Abstract

**Background/Objectives:** Bronchopulmonary dysplasia (BPD) frequently affects preterm infants and is associated with lasting morbidity. Early prediction remains challenging. The present study investigated whether hematological inflammatory markers—platelet-to-lymphocyte ratio (PLR), red cell distribution width (RDW), and red cell distribution width-to-platelet ratio (RPR)—can predict the development of BPD in preterm neonates. **Methods:** We performed a retrospective cohort study involving 100 infants born at less than 32 weeks’ gestation. Complete blood count (CBC) parameters were collected at birth, 72 h, 1 week, and 2 weeks of life. Associations between PLR, RDW, RPR, and BPD development were analyzed. Multivariate regression and receiver operating characteristic (ROC) curve analyses were carried out to evaluate the predictive performance of the markers. **Results:** Forty-nine percent of infants developed BPD. Those with BPD had significantly higher RDW, PLR, and RPR values, and lower lymphocyte and platelet counts at various time points. Gestational age, respiratory distress syndrome, and hematological indices independently predicted BPD. ROC analysis showed that RDW ≥ 67.2 and PLR ≥ 98.13 at 72 h, and RPR ≥ 0.3 at 7 and 14 days had good predictive performance. A combined scoring system, including clinical and hematological markers, achieved high sensitivity and specificity. **Conclusions:** Hematological inflammatory markers, especially RPR, PLR, and RDW, derived from routine CBC tests may serve as accessible, cost-effective tools for early BPD risk stratification in preterm infants. Additional studies are needed to confirm these results and better define their relevance in clinical practice.

## 1. Introduction

Bronchopulmonary dysplasia (BPD) was initially characterized by Northway and colleagues, and occurs in moderately preterm infants who are subjected to elevated airway pressures and increased oxygen levels due to long-term mechanical ventilation support, necessitated by severe respiratory distress syndrome (RDS). It is clinically characterized by hypoxemia and hypercapnia, often accompanied by cor pulmonale, and radiologically by increased density due to fibrosis on chest X-ray, as well as the presence of micro- and macrocysts, atelectasis and over-ventilated lung areas [1,2]. While the precise mechanisms that lead to lung development deterioration and permanent structural abnormalities in premature infants remain incompletely understood, key contributors to the development of BPD comprise surfactant deficiency, damage related to mechanical ventilation, oxygen-induced toxicity, pulmonary edema, inflammation, and impaired pulmonary vascular development (vasculogenesis) [3]. Early prediction of BPD remains clinically challenging due to the heterogeneous course of lung disease in preterm infants. Hematological inflammatory markers such as PLR, RDW, and RPR are easily obtainable from routine blood tests and may provide valuable early indicators to help identify infants at high risk, addressing this critical gap in neonatal care.

Pulmonary inflammatory processes are central to the mechanisms underlying BPD [4]. Both antenatal and postnatal infections, along with non-infectious factors such as free oxygen radicals, positive pressure ventilation, and an increase in lung altered circulation resulting from a persistent ductus arteriosus (PDA), can trigger the inflammatory response [5]. It has been shown that the tracheobronchial secretions of premature newborns infants progressing to BPD typically exhibit elevated concentrations of neutrophils, macrophages, leukotrienes, IL-6, IL-8, and TNF-α, which are indicators of inflammation [6]. Platelet (PLT), lymphocyte (L), and red cell distribution width (RDW) are individually recognized as systemic inflammatory markers. PLTs are actively involved in endothelial activation and the propagation of inflammatory responses. Lymphopenia reflects immune suppression and stress, both commonly seen in systemic inflammation. RDW, as a measure of anisocytosis, has been associated with oxidative stress, impaired erythropoiesis, and inflammatory cytokine activity. Together, these parameters provide valuable insight into the neonatal inflammatory status and have been studied as prognostic markers in various inflammatory and infectious conditions [7,8,9,10,11].

The red cell distribution width-to-platelet ratio (RPR) is a recently introduced, simple parameter that has shown strong predictive capacity in adult inflammatory disorders [7,8]. Furthermore, recent research has shown that it holds diagnostic significance in inflammatory processes among neonates [9,10]. While its application in bronchopulmonary dysplasia (BPD) is limited, evidence from these studies suggests that RPR may serve as a useful surrogate marker of systemic inflammation, supporting its evaluation in premature neonates at risk of BPD. A rise in platelet count together with reduced lymphocyte levels has been linked to inflammatory activity. The platelet-to-lymphocyte ratio (PLR) is derived from routine complete blood counts, determined by dividing platelet numbers by lymphocyte counts in peripheral blood. Considered a straightforward and easily accessible marker of systemic inflammation and immune response, the PLR is recognized as an independent prognostic factor for various conditions, including malignancies, cardiovascular disorders, sepsis, infectious diseases, and chronic obstructive pulmonary disease [11,12,13,14,15].

Given the contribution of inflammation to BPD pathogenesis, both RPR and PLR values are expected to be elevated in affected preterm infants. Beyond their individual associations, ratio-based indices such as PLR and RPR may enhance signal detection by integrating two biologically linked processes: platelet consumption/activation [16] and red cell size variability related to oxidative stress and impaired erythropoiesis [17]. Importantly, such ratios may also reduce the influence of systemic variability (e.g., hemodilution, sampling-related fluctuations), thereby amplifying clinically relevant signals compared with single parameters alone. Based on the current literature, no previous study has specifically evaluated the association between RPR and BPD in preterm infants. Therefore, this study provides novel insights by introducing RPR as a potential early biomarker for BPD, alongside the more widely studied PLR and RDW. We, therefore, hypothesized that PLR and RPR would outperform or at least complement single components (platelet count, RDW) for early BPD risk stratification.

## 2. Materials and Methods

We retrospectively reviewed a cohort of neonates admitted to Altunizade Acibadem Hospital, Istanbul, Turkey, during the period from January 2018 to January 2023. The study included preterm infants born before 32 weeks of gestation who were admitted to the neonatal intensive care unit (NICU) within six hours of birth. A total of 123 preterm infants with a gestational age < 32 weeks were initially assessed for eligibility in the NICU. Of these, 23 infants were excluded based on predefined criteria, including clinical or histological chorioamnionitis, PPROM, major congenital anomalies, and missing data. Finally, 100 infants were enrolled in the study. Both antenatal and postnatal risk factors for BPD were documented. Maternal history—including age, gravidity, and health status—was also collected. Data were collected on gestational age (GA), birth weight, sex, small-for-gestational-age (SGA) status, mode of delivery, Apgar scores, antenatal steroid exposure, use of exogenous surfactant, oxygen therapy, BPD severity, duration of mechanical ventilation, length of NICU stay, and neonatal comorbidities including respiratory distress syndrome (RDS), intraventricular hemorrhage (IVH), necrotizing enterocolitis (NEC), patent ductus arteriosus (PDA), retinopathy of prematurity (ROP), congenital pneumonia, and early-onset sepsis (EOS). BPD was diagnosed according to the criteria of the National Institute of Child Health and Human Development/National Heart, Lung, and Blood Institute (NICHD/NHLBI) consensus statement, and assessed at 36 weeks’ postmenstrual age [18]. EOS was defined as sepsis within the first 72 h of life, and was considered suspected when at least two clinical and two laboratory findings were present [19]. A positive blood culture in these infants was regarded as confirming EOS. Clinical manifestations were categorized as follows: (1) respiratory instability (apnea, tachypnea, increased oxygen demand, or need for ventilatory support); (2) cardiovascular instability (bradycardia, tachycardia, arrhythmia, urine output < 1 mL/kg/h, hypotension, mottled skin, or poor peripheral perfusion); (3) altered body temperature (hypothermia, hyperthermia, or temperature fluctuations); (4) gastrointestinal disturbances (feeding intolerance, weak sucking, or abdominal distension); (5) skin and subcutaneous findings (petechiae or sclerema); and (6) nonspecific signs (irritability, lethargy, or hypotonia). Laboratory indicators included the following: (1) WBC count < 4000 × 10^9^/L or >20,000 × 10^9^/L; (2) immature-to-total neutrophil ratio (I/T) > 0.2; (3) PLT count < 100,000 × 10^9^/L; (4) CRP > 15 mg/L or PCT ≥ 2 ng/mL; (5) repeated glucose abnormalities, including hyperglycemia (>180 mg/dL) or hypoglycemia (<45 mg/dL); and (6) metabolic acidosis defined as base excess < −10 mEq/L or serum lactate > 2 mMol/L [19]. RDS was diagnosed based on the clinical signs of respiratory distress and radiographic evidence, requiring supplemental oxygen or ventilatory support [20]. IVH was classified according to Papile criteria (grade I–IV) using cranial ultrasonography [21]. ROP was staged using the International Classification of Retinopathy of Prematurity, revised in 2005 [22]. PDA was diagnosed by echocardiographic evidence of a hemodynamically significant ductus arteriosus, according to Turkish Neonatal Society guidelines [23]. NEC was staged based on modified Bell criteria [24]. The diagnostic definitions for SGA, PROM, and congenital pneumonia were adopted from established criteria reported in earlier studies [25,26,27]. Infants who were intubated were managed on mechanical ventilation using synchronized intermittent mandatory ventilation (SIMV) mode. High-frequency ventilation was also applied if clinically indicated. They were extubated as soon as possible and followed up with nasal intermittent positive pressure ventilation (nIPPV). The surfactant used in this study was poractant alpha (Curosurf; Chiesi, Cary, NC, USA), an extract of natural porcine lung consisting of surfactant. Surfactant therapy was administered at an initial dose of 200 mg/kg, with subsequent doses of 100 mg/kg given as clinically indicated according to NICU protocols. Hematological parameters, including PLT count, absolute L count, and RDW were recorded at birth, 72 h, and at 1 and 2 weeks of life. The postnatal time points (72 h, day 7, and day 14) were selected to capture early hematologic and inflammatory changes relevant to BPD development, in line with prior neonatal studies and standard clinical monitoring practices. The PLR was calculated as the PLT count divided by the absolute lymphocyte count, while the red cell distribution width-to-platelet ratio (RPR) was obtained by dividing the RDW value by the PLT count.

We estimated that we would need 100 patients for the comparison of two independent groups (effect size d: 0.8, alpha error: 0.05, 1-beta error: 0.99). Although a formal power analysis is not strictly necessary in a retrospective study with a fixed sample size, we provided this calculation to demonstrate that the available cohort exceeded the minimum required number of subjects, thereby supporting the adequacy of our sample.

Ethical approval for this study was obtained from the Acibadem University School of Medicine Ethics Committee (approval number: 2023-08/295), and written informed consent was provided by the parents of all participants.

### Statistical Analysis

We presented data with the mean ± standard deviation, median (interquartile range), and % (*n*), and used Kolmogorov–Smirnov test to assess normality with skewness, curtosis, and Q–Q plots. To compare 2 independent groups, we used Student’s *t*-test or the Mann–Whitney U test by normality assumption. We also used Welch’s *t*-test to compare normal but non-homogenously distributed (tested with Levene’s test) two groups. Also, we used the Chi-square test of Fisher exact test by expected counts to compare 2 groups containing nominal data.

We used Kendall’s tau test to assess correlation coefficients; afterward, we put the correlated factors as the possible confounders into a multivarite model (generalized linear model, GLM) considering multicollinearity by variance inflating factor. We used the forward selection and stepwise selection methods to asesss the most significant factors, and presented loglikelihood ratio results (χ^2^, and *p*). Finally, we presented the correlation strength with R^2^. We built receiver operating characteristic (ROC) curves with the significant confounders. We used JAMOVI 2.3.18 statistical package program with PPDA extension. The alpha error rate of *p* = 0.05 was considered significant for comparisons and 0.10 for assessing confounders in multivariate analysis. It should be noted that GA was analyzed in a restricted cohort (<32 weeks), already representing a high-risk population for BPD. This limited variability explains the unexpectedly low AUC of GA when considered as a single predictor. In broader, population-based cohorts, GA typically demonstrates higher predictive performance, but in our NICU-based sample, its discriminative ability was attenuated. This context is important for interpreting the AUC values of the significant predictors.

## 3. Results

We included 100 patients in the study (median gestational age: 29.0 [28.0–30.3] weeks; mean birth weight: 1290 ± 320 g; 44% male, 56% female). BPD was diagnosed in 49% of the infants, while 51% had no BPD. The high BPD rate (49%) in our cohort reflects the inclusion of very preterm infants (<32 weeks GA) with a high incidence of respiratory distress syndrome (RDS) and limited antenatal steroid exposure, consistent with standard NICHD/NHLBI diagnostic criteria assessed at 36 weeks postmenstrual age. The descriptive characteristics and intergroup comparisons (BPD vs. non-BPD) are presented in Table 1. Infants with BPD had a significantly lower gestational age, birth weight, and APGAR scores. By contrast, infants in the BPD group had significantly longer durations of oxygen therapy, invasive and non-invasive mechanical ventilation, and higher incidences of IVH, NEC, ROP, and PDA.

Lymphocyte counts were significantly lower in the BPD group at birth, 72 h, and at the first week. PLT levels were significantly lower at the first and second weeks. RDW was consistently higher in the BPD group across all time points. PLR was higher at birth and at 72 h, while RPR was elevated at 72 h, and during the first and second weeks. Detailed comparisons of CBC parameters are shown in Table 2.

Correlation analyses showed that lower gestational age (r = 0.544, *p* < 0.001), lower birth weight (r = 0.473, *p* < 0.001), and lower APGAR scores were associated with BPD. Additionally, lymphocyte counts were negatively correlated with BPD at all time points. PLT showed negative correlation at 72 h, the first week, and second week. RDW was positively correlated with BPD at all time points. PLR showed positive correlation at birth and at 72 h, and RPR at 72 h, first, and second weeks (Table 3).

Multivariate (GLM) models were constructed to assess the predictors of BPD at each time point, incorporating gestational age (GA), birth weight, surfactant requirement, PDA (after the 72nd hour calculations), EOS, and CBC parameters. EOS was not found to be a significant confounder in any model.

**Figure 1:**
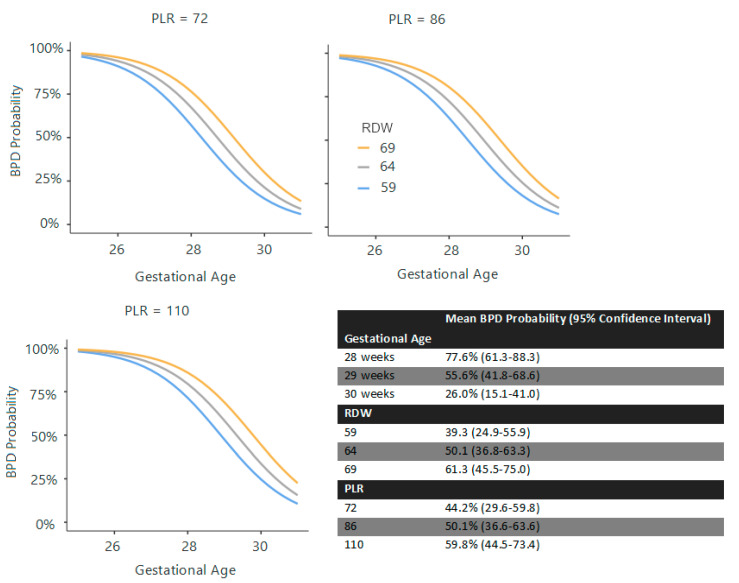
Estimated marginal means and curves regarding BPD prediction at the 72nd hour. Note: This graph and its corresponding estimated marginal means table represent the estimated probability of BPD based on gestational age, RDW, and PLR. Surfactant requirement was omitted from the curves for clarity, as all patients who received surfactant developed BPD.

**Figure 2:**
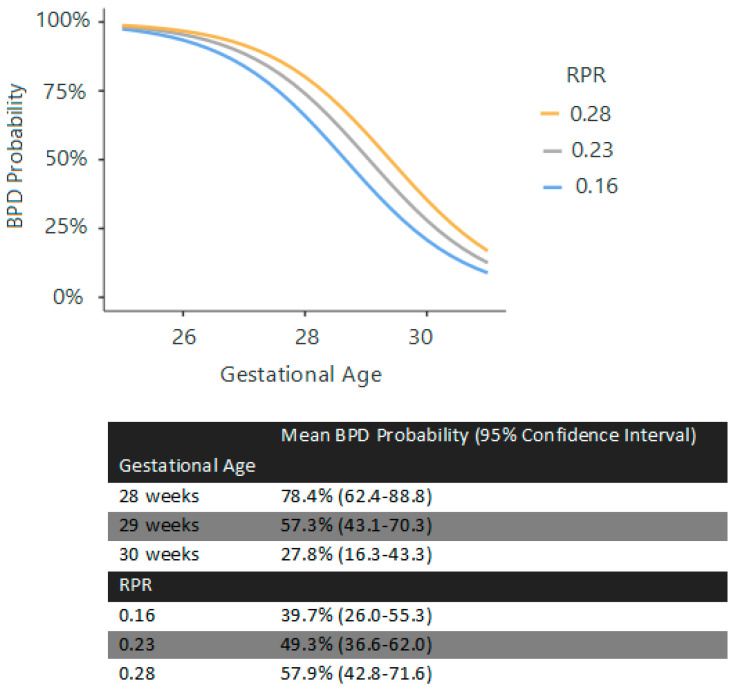
Estimated marginal means and the curves regarding BPD prediction at 7th day. Note: This graph and its corresponding estimated marginal means table represent the estimated probability of BPD based on gestational age and RPR. Surfactant requirement was omitted from the curves for clarity, as all patients who received surfactant developed BPD.

**Figure 3:**
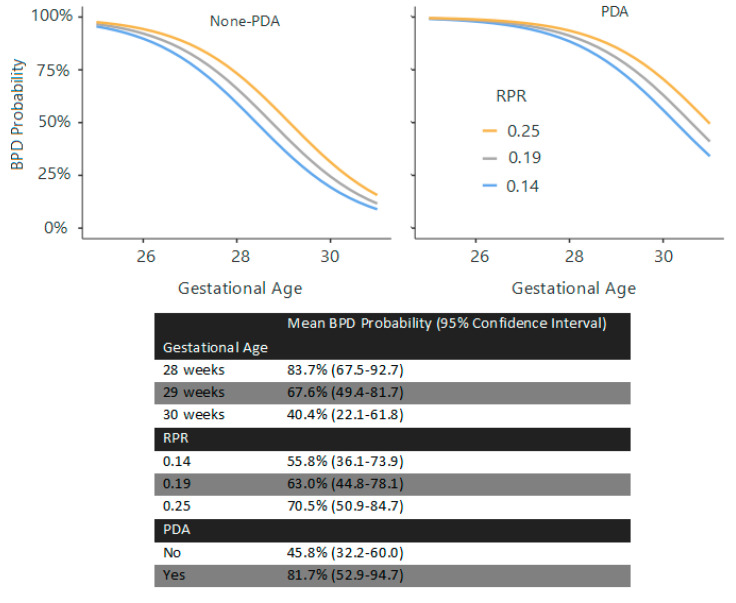
Estimated marginal means and the curves regarding BPD prediction at 14th day. Note: This graph and its estimated marginal means table represent the estimated probability of BPD based on gestational age and RPR. Surfactant requirement was omitted from the curves for clarity, as all patients who received surfactant developed BPD.

At birth, GA (χ^2^ = 9.07), birth weight (χ^2^ = 6.18, *p* = 0.013), and surfactant requirement (χ^2^ = 7.17, *p* = 0.007) were significant predictors (R^2^ = 0.404). At 72 h, GA (χ^2^ = 27.09, *p* < 0.001), surfactant requirement (χ^2^ = 6.88, *p* = 0.009), PLR (χ^2^ = 3.41, *p* = 0.065), and RDW (χ^2^ = 6.75, *p* = 0.009) were predictive (R^2^ = 0.441) (Figure 1). At the first week, GA (χ^2^ = 26.5, *p* < 0.001), surfactant requirement (χ^2^ = 8.69, *p* = 0.003), and RPR (χ^2^ = 7.36, *p* = 0.007) were significant (R^2^ = 0.413) (Figure 2). At the second week, GA (χ^2^ = 19.31, *p* < 0.001), surfactant requirement (χ^2^ = 6.90, *p* = 0.009), PDA (χ^2^ = 4.05, *p* = 0.044), and RPR (χ^2^ = 3.31, *p* = 0.069) were the predictors (R^2^ = 0.413) (Figure 3).

ROC analysis revealed that GA ≥ 29 weeks predicted absence of BPD (not prediction of BPD) with 88.2% sensitivity and 65.3% specificity (AUC: 0.845 for prediction of non-BPD, AUC:0.155 for prediction of BPD). PLT on day 14 could not efficiently predict BPD in ROC analysis (AUC: 0.625). RDW ≥ 67.2 at 72 h, PLR ≥ 98.13 at 72 h, and RPR ≥ 0.3 at the first and second weeks were optimal thresholds for predicting BPD (Table 4). One point was assigned for each marker exceeding these thresholds (or falling below GA < 29 weeks).

**Figure 4:**
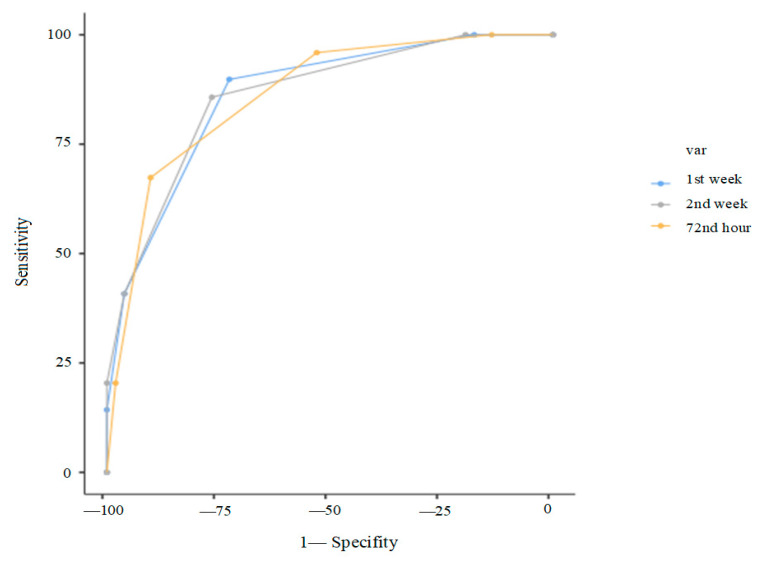
ROC curves of the new basic predictive scoring models. Note: Thresholds for the predictive scoring models were defined as follows: at 72 h, four points corresponded to GA < 29 weeks, surfactant requirement, RDW ≥ 67.2, and PLR ≥ 98.13; at 7 days, three points corresponded to GA < 29 weeks, surfactant requirement, and RPR ≥ 0.3; and at 14 days, four points corresponded to GA < 29 weeks, surfactant requirement, PDA, and RPR ≥ 0.3.

Finally, composite scoring systems were evaluated. At 72 h, a score of ≥2 out of 4 (GA < 29 weeks, surfactant requirement, RDW ≥ 67.2, PLR ≥ 98.13) yielded 95.9% sensitivity, and a score of ≥3 gave 90.2% specificity (AUC: 0.86). At day 7, a score of ≥2 out of 3 (GA < 29 weeks, surfactant requirement, RPR ≥ 0.3) yielded 85.7% sensitivity; a score of 3/3 gave 100% specificity (AUC: 0.85). At day 14, a score of ≥2 out of 4 (GA < 29 weeks, surfactant requirement, RPR ≥ 0.3) provided 81.6% sensitivity and 78.4% specificity; 4/4 yielded 100% specificity (AUC: 0.85). (Table 5, Figure 4).

## 4. Discussion

The present study highlights the significant association between early hematological parameters—specifically PLR, RDW, and RPR—and the development of BPD in preterm infants. Our findings support the notion that systemic inflammation significantly contributes to BPD development, and that readily available hematoloindices may serve as practical and cost-effective surrogate markers. While previous studies have explored individual markers related to inflammation and BPD risk, our study is among the first to comprehensively evaluate RPR alongside PLR and RDW, revealing their dynamic changes over the first two weeks of life and their predictive utility. These results not only align with but also expand upon existing literature, offering new insights into early risk stratification and potential avenues for intervention in vulnerable neonatal populations.

Platelets play a central role in neonatal inflammation and vascular development, both critical in the pathogenesis of BPD [26,27]. In our study, PLT counts were notably lower in the BPD group during the first and second postnatal weeks. Concurrently, RDW was significantly elevated at all timepoints, and RPR was markedly increased from the first postnatal week onward. These findings suggest that PLTs may be consumed in pulmonary microvascular injury and inflammation. Our findings are in agreement with Yan et al., who observed that preterm infants with BPD showed reduced PLT counts and higher levels of PLT-related markers, including CD62P and thrombopoietin [28]. Similarly, Wang et al. identified PLT counts ≤ 177 × 10^9^/L at day 14 as a strong predictor for BPD [29]. Incorporating RPR into our predictive model further enhanced its diagnostic value, particularly during the first two postnatal weeks.

We observed that RDW and PLR values at birth were significantly elevated in infants who later developed BPD, while PLT counts did not show a significant difference at this timepoint. Additionally, L counts were lower in the BPD group. These results are summarized in Table 3. Our findings partially align with those of Chen et al., who reported higher neutrophil, monocyte, and PLT counts in infants with BPD at birth [30]. The discrepancy in platelet counts may be attributable to differences in gestational age distributions or variations in BPD severity among study cohorts. Our results suggest that early alterations in red cell morphology and lymphocyte-mediated immunity, as reflected by RDW and PLR, may precede marked PLT consumption.

Gestational age (GA), birth weight, surfactant requirement, and patent ductus arteriosus (PDA) were among the strongest predictors of bronchopulmonary dysplasia (BPD) in our cohort, consistent with prior literature [31,32]. Importantly, multivariate generalized linear models (GLM) combining clinical and hematologic variables demonstrated improved predictive accuracy. For instance, a predictive model including GA, surfactant requirement, PLR, and RDW at 72 h of life yielded an area under the curve (AUC) of 0.86, demonstrating 67.4% sensitivity and 90.2% specificity. These findings align with Tao et al., who identified low GA, low birth weight, prolonged oxygen therapy, and PDA as major predictors of BPD in a large cohort of neonates with RDS (n = 625) [31]. Similarly, Maytasari et al. reported that mechanical ventilation exceeding two days and PDA were significant risk factors [32]. Our models parallel these established predictors but advance the field by integrating hematologic biomarkers, thereby refining early risk estimation in preterm infants. The scoring system we developed allows for flexible and time-specific risk stratification using routine complete blood count (CBC) parameters, enhancing clinical utility. Furthermore, our time-point-specific scoring systems demonstrated strong predictive performance. At 72 h, the model combining GA < 29 weeks, surfactant requirement, RDW ≥ 67.2, and PLR ≥ 98.13 yielded 95.9% sensitivity when ≥2 criteria were met and 90.2% specificity with ≥3 criteria were met (AUC: 0.86). Similarly, the day 7 model incorporating GA, surfactant requirement, and RPR ≥ 0.3 provided 85.7% sensitivity with 2 points and 100% specificity with 3 points (AUC: 0.85). The day 14 model, including GA, surfactant requirement, and RPR ≥ 0.3, achieved 100% specificity when all four criteria were present. These scoring systems offer practical, stepwise tools for clinicians to estimate BPD risk early and accurately using routinely available parameters.

Systemic inflammation is a key contributor to BPD development [4,5]. In our study, infants who eventually developed BPD exhibited significantly higher PLR values from birth, reaching a peak at 72 h of life. Moreover, RPR showed a progressive increase over the first two weeks of life. These indices likely reflect systemic inflammatory responses, including lymphopenia, thrombocytopenia, and red cell dysregulation. Previous studies by Jiang et al. and Cao et al. similarly reported that the neutrophil-to-lymphocyte ratio (NLR) and PLR levels within 72 h were significantly higher in neonates who subsequently developed BPD [33,34]. Our findings support this early inflammatory signature and extend it by demonstrating a time-dependent trajectory of RDW and RPR that is associated with BPD risk.

RDW, a marker of anisocytosis and oxidative stress, was consistently elevated in BPD infants from birth through the second postnatal week. The RPR, which integrates RDW and PLT count, showed moderate predictive performance with AUC values ranging from 0.67 to 0.69. To our knowledge, this study is the first to longitudinally evaluate RPR in preterm neonates at risk for BPD. While prior studies have assessed inflammatory markers such as PLR and NLR, RPR has not been examined in the context of BPD. This novel contribution broadens the spectrum of hematological indices with potential diagnostic relevance in neonatal lung disease. The early rise in RPR likely reflects endothelial stress, aberrant marrow responses, and systemic inflammation. Given its derivation from standard CBC parameters, RPR represents a simple, cost-effective biomarker for potential inclusion in NICU risk assessment algorithms. Although PLR and RPR were significantly associated with subsequent BPD, the diagnostic performance of these indices was modest (AUCs 0.65–0.69), indicating that they should not be considered standalone predictive tools. Instead, their value may lie in complementing established clinical predictors such as gestational age and respiratory course, to refine early risk stratification. Future research should investigate whether incorporating these indices into multiparametric models can improve predictive accuracy.

Beyond their traditional role in hemostasis, PLTs actively participate in inflammation, angiogenesis, and immune signaling [28,35]. The lower PLT counts observed in our BPD group during the first two postnatal weeks are consistent with hypotheses of pulmonary PLT consumption and impaired megakaryopoiesis in response to lung injury. Yan et al. documented increased circulating megakaryocytes and thrombopoietin (TPO) levels in BPD infants, supporting a model of enhanced PLT turnover triggered by pulmonary damage [28]. Our findings complement this by illustrating how dynamic changes in RPR and PLR reflect the evolving hematologic–inflammatory interface. These markers, indicative of both systemic and pulmonary inflammation, may provide prognostic value and inform the timing of therapeutic interventions. Notably, the combined assessment of PLR and RPR improved predictive accuracy, as their dynamic changes at 72 h and during the first two postnatal weeks captured complementary aspects of systemic inflammation and hematologic stress.

Integrating hematologic indices with established clinical risk factors can improve the early prediction of BPD during the critical postnatal period. Our data indicate that CBC-derived parameters such as PLR, RPR, and RDW, measured at 72 h and throughout the first two postnatal weeks, can effectively stratify risk alongside GA, surfactant requirement, and PDA. The temporal changes in these markers may help clinicians identify high-risk preterm infants early, potentially guiding decisions on respiratory support intensity, timing of corticosteroid therapy, or other targeted interventions. Nevertheless, prospective studies are needed to validate the integration of these markers into real-time clinical decision-making.

These biomarkers are cost-effective, easily obtainable, and suitable for incorporation into bedside clinical tools, potentially guiding early intervention strategies. Importantly, this study is the first to highlight the utility of RPR as a predictive biomarker for BPD in preterm neonates. This novel finding expands the range of hematological indices with potential diagnostic relevance in neonatal lung disease. Applied to patient monitoring, optimization of corticosteroid therapy, or individualized ventilator weaning, these markers represent a practical advance in NICU care workflows.

In clinical practice, the early recognition of elevated PLR, RDW, or RPR values may support neonatologists in optimizing the timing of therapeutic interventions. For example, identifying infants at higher risk for BPD using these readily available hematological markers could prompt earlier consideration of corticosteroid therapy, closer monitoring of ventilatory requirements, or timely adjustments in respiratory support. Integrating these markers into NICU decision-making algorithms may, therefore, improve outcomes by ensuring that preventive and therapeutic measures are implemented at the most appropriate time.

Certain exclusion criteria, such as PROM and clinical or histological chorioamnionitis, may limit the generalizability of our findings. As these conditions are the common causes of preterm delivery, and excluding affected infants could result in a study population that is not fully representative of all preterm neonates < 32 weeks GA.

There are additional limitations to this study, including its retrospective nature and single center setting which may restrict the generalizability of the findings. Data on cytokines or lung-specific biomarkers were not available. Furthermore, although we proposed time-point-specific scoring systems, prospective validation is needed to confirm their clinical applicability. Additionally, some individual cut-off values, particularly for PLR, demonstrated modest AUCs with lower sensitivity or specificity when considered alone, suggesting that their predictive value may be limited without integration into broader multivariate or scoring models.

We acknowledge that medical interventions, including antibiotics, blood transfusions, and corticosteroid therapy, may have influenced hematological parameters such as platelet counts and inflammatory markers. Due to the retrospective design, the impact of these potential confounders could not be fully controlled, which should be considered when interpreting our results. Unmeasured confounders, missing data, and center-specific practices may also have affected the observed associations, highlighting the need for future multicenter prospective studies. Such studies should focus on multicenter validation, mechanistic correlation with cytokine profiles, and the development of real-time risk calculators integrating CBC markers with clinical and radiologic parameters. Moreover, integrating hematological markers (PLR, RDW, RPR) with clinical variables such as gestational age, birth weight, severity of respiratory support, and radiologic findings may further enhance predictive accuracy and facilitate the development of more comprehensive BPD risk assessment models. If validated, PLR and RPR could play a central role in early decision-making in neonatal lung care.

Our analysis treated BPD as a binary outcome and did not stratify by severity (mild, moderate, severe). It is possible that biomarker trends vary according to BPD severity, and future studies should explore severity-specific changes to provide more precise guidance for clinical risk stratification.

## 5. Conclusions

In conclusion, PLR, RDW, and RPR derived from routine blood counts may serve as practical and low-cost adjuncts for the early risk stratification of BPD in preterm infants. While their individual diagnostic contribution was modest (AUC ~0.65–0.69) and remained consistent across different time points, predictive accuracy improved substantially when these markers were combined with established predictors such as gestational age, surfactant therapy, and PDA (composite model AUC ~0.81–0.86). These findings underscore that hematologic indices should not be considered standalone predictors but can provide complementary value when integrated into composite risk models. Incorporating such parameters into NICU risk assessment protocols may facilitate a more refined identification of high-risk infants. Future multicenter prospective studies are warranted to validate these results, and develop standardized approaches for BPD prevention and management.

## Figures and Tables

**Table 1:** Descriptive characteristics and comparison between preterm infants with and without bronchopulmonary dysplasia (BPD).

	Non-BPD (n = 49)	BPD (n = 51)	*p*		Non-BPD (n = 49)	BPD (n = 51)	*p*
Maternal age	31.9 ± 4.5	33.6 ± 4.5	0.057 ^S^	Delivery	98.0% (n = 50)	100.0% (n = 49)	1 ^F^
Gestation age	30 (30–31)	28 (27–29)	<0.001 ^M^	Gender	58.8% (n = 30)	53.1% (n = 26)	0.562 ^C^
Birth weight	1565 (1250–1698)	1100 (920–1240)	<0.001 ^M^	SGA	2.0% (n = 1)	6.1% (n = 3)	0.357 ^F^
Duration of oxygen therapy	10 (7–18)	45 (38–55)	<0.001 ^M^	PROM	2.0% (n = 1)	6.1% (n = 3)	0.357 ^F^
Duration of NIV therapy	108 (81–181)	642 (426–764)	<0.001 ^M^	Antenatal steroid	49.0% (n = 25)	57.1% (n = 28)	0.416 ^C^
Duration of SIMV therapy	0 (0–21)	26 (0–124)	<0.001 ^M^	Endotracheal surfactant	78.4% (n = 40)	100% (n = 49)	<0.001 ^F^
Duration of NICU	32 (28–42)	62 (54–67)	<0.001 ^M^	IVH	3.9% (n = 2)	28.6% (n = 14)	<0.001 ^C^
APGAR 1	6.0 (4.0–7.0)	5.0 (3.0–6.0)	0.033 ^M^	NEC	5.9% (n = 3)	22.4% (n = 11)	0.017 ^C^
APGAR 5	8.0 (7.5–8.0)	7.0 (7.0–8.0)	0.011 ^M^	ROP	5.9% (n = 3)	42.9% (n = 21)	<0.001 ^C^
				PDA	5.9% (n = 3)	36.7% (n = 18)	<0.001 ^C^

^S^: Student’s *t* test, ^M^: Mann–Whitney U test, ^C^: Chi-square test, ^F^: Fisher’s exact test. NIV: non-invasive ventilation, SIMV: synchronized intermittent mandatory ventilation, NICU: neonatal intensive care unit, SGA: small for gestational age, IVH: intraventricular hemorrhage, NEC: necrotising enterocolitis, ROP: retinopathy of prematurity, PDA: patent ductus arteriosus. PROM was assessed in the screened population but excluded from the final enrolled cohort per study criteria.

**Table 2:** Comparison of complete blood count (CBC) parameters between BPD and non-BPD groups at various time points.

	No BPD (n = 49)	BPD (n = 51)	*p*	No BPD (n = 49)	BPD (n = 51)	*p*
	At birth			At 1st week (7th day)		
Lymphocyte	4.5 ± 1.8	3.6 ± 1.4	0.009 ^S^	4.5 ± 1.3	3.6 ± 1.2	<0.001 ^S^
Platelet	251.2 ± 70.0	240.1 ± 68.1	0.423 ^S^	304.8 ± 93.2	258.5 ± 107.6	<0.001 ^S^
RDW	64.6 ± 7.2	69.8 ± 10.3	0.004 ^W^	58.8 ± 6.0	66.1 ± 11.1	<0.001 ^W^
PLR	55.1 (42.7–77.3)	67.7 (57.6–89.3)	0.023 ^M^	71.8 ± 25.6	73.2 ± 25.1	0.777 ^S^
RPR	0.3 ± 0.1	0.3 ± 0.1	0.187 ^S^	0.2 ± 0.1	0.3 ± 0.2	<0.001 ^W^
	At 72nd h			At 2nd week (14th day)		
Lymphocyte	3.0 ± 0.9	2.4 ± 1.3	0.008 ^W^	5.2 ± 1.3	4.7 ± 1.5	0.120 ^S^
Platelet	240.3 ± 70.1	214.3 ± 81.0	0.089 ^S^	355.0 ± 107.8	306.1 ± 121.1	0.035 ^S^
RDW	61.8 ± 6.3	68.8 ± 10.9	<0.001 ^W^	58.0 ± 6.0	65.5 ± 8.5	<0.001 ^W^
PLR	83.4 ± 24.0	106.2 ± 52.5	0.007 ^W^	71.7 ± 23.8	67.9 ± 24.9	0.439 ^S^
RPR	0.25 (0.21–0.33)	0.30 (0.25–0.45)	0.015 ^M^	0.18 ± 0.06	0.25 ± 0.14	<0.001 ^W^

^S^: Student’s *t*-test, ^M^: Mann–Whitney U test, ^W^: Welch’s *t*-test. Lymphocyte: lymphocyte count, Platelet: platelet count, RDW: red cell distribution width, PLR: platelet-to-lymphocyte ratio, RPR: red cell distribution width-to-platelet ratio.

**Table 3:** Correlation coefficients of CBC parameters with BPD at birth, 72nd hour, 1st week, and 2nd week.

	Birth	72nd h	1st Week	2nd Week
Lymphocyte	−0.179 *	−0.273 ***	−0.269 *	−0.104 *
Platelet	−0.087	−0.163 *	−0.163 *	−0.178 *
RDW	0.212 *	0.286 ***	0.321 ***	0.369 ***
PLR	0.188 *	0.207 *	0.044	−0.067
RPR	0.128	0.203 *	0.244 **	0.268 **

Kendall’s tau test. *: *p* < 0.05, **: *p* < 0.01, ***: *p* > 0.001. Lymphocyte: lymphocyte count, Platelet: platelet count, RDW: red cell distribution width, PLR: platelet-to-lymphocyte ratio, RPR: red cell distribution width-to-platelet ratio.

**Table 4:** Receiver operating characteristic (ROC) analysis of hematological markers for predicting BPD.

	Cutpoint	Sensitivity (%)	Specificity (%)	PPV (%)	NPV (%)	AUC
72nd h RDW	65.6	59.18%	74.51%	69.05%	65.52%	0.70
	66.5	55.10%	78.43%	71.05%	64.52%	0.70
	*67.2*	*53.06*%	*82.35*%	*74.29*%	*64.62*%	*0.70*
72nd h PLR	84.7	65.31%	58.82%	60.38%	63.83%	0.65
	*98.13*	*51.02*%	*74.51*%	*65.79%*	*61.29%*	*0.65*
	98.88	48.98%	76.47%	66.67%	60.94%	0.65
1st week RPR	0.26	46.94%	80.39%	69.70%	61.19%	0.67
	0.28	40.82%	88.24%	76.92%	60.81%	0.67
	*0.3*	*36.73*%	*92.16*%	*81.82%*	*60.26*%	*0.67*
2nd week RPR	0.27	38.78%	92.16%	82.61%	61.04%	0.69
	0.29	32.65%	98.04%	94.12%	60.24%	0.69
	*0.3*	*30.61*%	*100*%	*100*%	*60.00*%	*0.69*

RDW: red cell distribution width, PLR: platelet-to-lymphocyte ratio, RPR: red cell distribution width-to-platelet ratio. Note: Italicized values represent the selected thresholds.

**Table 5:** Performance of combined predictive scoring models for BPD risk stratification at different postnatal time points.

Time Point	Predictor	Cutpoint	Sensitivity (%)	Specificity (%)	PPV (%)	NPV (%)	AUC
72nd h	GA < 29 weeks Surfactant + RDW ≥ 67.2 + PLR ≥ 98.13	2	95.92%	52.94%	66.20%	93.10%	0.86
		3	67.35%	90.20%	86.84%	74.19%	0.86
		4	20.41%	98.04%	90.91%	56.18%	0.86
7th day	GA < 29 weeks Surfactant + RPR ≥ 0.3	1	100%	17.65%	53.85%	100%	0.85
		2	85.71%	72.55%	75.00%	84.09%	0.85
		3	30.61%	100%	100%	60.00%	0.85
14th day	GA < 29 weeks Surfactant + PDA+ RPR ≥ 0.3	1	100%	19.61%	54.44%	100%	0.86
		2	85.71%	76.47%	77.78%	84.78%	0.86
		3	40.82%	96.08%	90.91%	62.82%	0.86
		4	20.41%	100%	100%	56.67%	0.86

GA: gestational age, Surfactant: surfactant requirement, RDW: red cell distribution width, PLR: platelet-to-lymphocyte ratio, RPR: red cell distribution width-to-platelet ratio. The cutpoints represent how many predictors were violated. Note: As an example, in an infant born < 29 weeks, who underwent surfactant therapy and with a RPR ≥ 0.3 at 7th day (three points over 3) or 14th day (four points over 4), the specificity is 100%.

## Data Availability

The data presented in this study will be shared upon reasonable requests from the corresponding author but cannot be shared in public as authors have no permission granted by the ethical board.
